# Psychosocial interventions for dementia in low- and middle-income countries (LMICs): a systematic review of effectiveness and implementation readiness

**DOI:** 10.1080/13607863.2019.1695742

**Published:** 2019-12-09

**Authors:** Charlotte R. Stoner, Monisha Lakshminarayanan, Helen Durgante, Aimee Spector

**Affiliations:** a Research Department of Clinical Educational and Health Psychology, University College London (UCL), London, UK; b Dementia Care in Schizophrenia Research Foundation (DEMCARES in SCARF), Chennai, Tamil Nadu, India; c Department of Psychiatry, Federal University of São Paulo (UNIFESP), São Paulo, Brazil

**Keywords:** Developing countries, Alzheimer’s disease, cognition, quality of life

## Abstract

**Introduction:**

Despite wide uptake in high-income countries (HICs), less is known about the effectiveness and implementation of psychological, social and cognitive interventions in low- and middle-income countries (LMICs). Despite this, such interventions are increasingly used. The aim of this review was to appraise the effectiveness and implementation readiness of psychosocial interventions for people with dementia in LMICs.

**Methods:**

A systematic search of databases from 1998–2019. Studies were rated on two scales assessing quality and implementation readiness.

**Results:**

Seventeen articles describing 11 interventions in six countries were evaluated. Interventions included Cognitive Stimulation Therapy (CST), a Multidisciplinary Cognitive Rehabilitation Programme (MCRP), singing interventions, occupational therapy and reminiscence therapy. The quality of included studies was variable, and many had low sample sizes. Evidence for improving both cognition and quality of life was found in two interventions: Cognitive Stimulation Therapy (CST) and a Multidisciplinary Cognitive Rehabilitation Programme (MCRP). Implementation issues were more likely to be explored in studies of Cognitive Stimulation Therapy (CST) than in any other intervention.

**Conclusions:**

Of the included studies here, CST appears to be the most implementation ready, improving both cognition and quality of life with implementation readiness effectively explored in two LMIC countries: India and Tanzania.

## Introduction

There are currently 27.3 million people living with dementia in low- and middle-income countries (LMICs), with this number forecast to increase substantially and at a greater rate than in high-income countries (HICs) over the next 30 years (Alzheimer's Disease International, 2015). However, awareness of dementia in LMICs can be low and there is often a diagnosis and treatment gap. For example, it is estimated that only 20% of people with dementia in Brazil (Nakamura, Opaleye, Tani, & Ferri, 2015) and 5% of people with dementia in India have received a diagnosis (Prince, Livingston, & Katona, 2007).

In LMICs, there are often no routinely offered non-pharmacological treatment options including cognitive, psychological and social interventions (Alzheimer's Disease International, 2016). Psychosocial interventions are now firmly established as an alternative or supplementary treatment option in HIC countries including the United Kingdom (UK). Recent awards by UK and European funding bodies have included interventions designed to promote independence (Csipke et al., 2018; Wenborn et al., 2016) and improve wellbeing and health (Whitaker et al., 2014), all with psychosocial interventions. Results of such studies have been varied and, despite being widely funded, psychosocial interventions for people with dementia often fail to be ‘scaled up’ or implemented in health services. An exception to this is Cognitive Stimulation Therapy (CST), which has been implemented in approximately 90% of UK Memory Services (Royal College of Psychiatrists, 2016).

As access to services and pharmacological treatment in LMICs are variable, with no specialist care available in some sub-Saharan African countries like Tanzania (Paddick et al., 2015), psychosocial interventions may be an important means of improving symptoms of Alzheimer’s disease and other dementias. However, despite their wide use in HICs, less is known about their effectiveness and the degree to which these interventions have been implemented in LMICs.

The aims of this systematic review were to:

1. Identify all psychological, social and cognitive interventions in use for people with dementia in LMICs.
2. Appraise the quality of these studies using an established checklist.
3. Assess the effectiveness of interventions in each country.
4. Assess how implementation ready each intervention was.
5. Make recommendations for appropriate and effective psychosocial interventions for people with dementia in LMICs.

## Methods

### Design

A systematic search of published literature on interventions delivered to people with dementia in LMICs over the preceding 10 years was conducted. The term psychosocial was used to encompass all psychological, social and cognitive interventions. In addition to appraising the effectiveness of these interventions, implementation issues such as barriers to or facilitators of delivering interventions in each country were sought. The preceding 10 years was selected to ensure that evidence sourced regarding implementation issues was still relevant. Systematic principles were followed for searching, screening and appraising results (Moher, Liberati, Tetzlaff, Altman, & the PRISMA Group, 2009).

### Search strategy

Cochrane Controlled Register of Trials (CENTRAL), PubMed, EMBASE, PsycINFO and MEDLINE were searched for studies published between 2008 and June 2019. Search terms were developed using terms and interventions identified in a previous reviews (McDermott, Charlesworth et al., 2018), a list of LMIC search terms developed by Cochrane Groups for CENTRAL and based on World Bank data, and previous reviews focusing on people with dementia (Woods, Aguirre, Spector, & Orrell, 2012).

Examples of search terms for interventions included: ‘non-pharmocolog*’ or ‘non-drug’ or ‘psychosocial’ or ‘psycholog* therap*’ or ‘social* intervention’ or ‘social* therapy’ or ‘occupational therapy’ or OT or psychoeducation or psycho-education or educat* or support or psychotherapy or ‘cognitive behavioural therapy’ or ‘cognitive rehabilitat*’ or ‘cognitive stimulation therapy’ or CST or ‘group therapy’.

Examples of search terms used to identify LMICs included: Africa or Asia or Caribbean or ‘West Indies’ or ‘South America’ or ‘Latin America’ or ‘Central America’ or Macedonia or Madagascar or ‘Malagasy Republic’ or Malaysia or Malaya or Malay or Sabah or Sarawak or developing countr*’ or ‘under developed countr*’ or ‘underdeveloped countr*’ or ‘middle income countr*’ or MIC or LMIC or LIC or ‘low* income countr*’.

Finally, examples of terms used to identify people with dementia consisted of: Dement* or Alzheimer or ‘vascular dementia’ or ‘cognitive impairment’ or ‘Huntington’s disease’ or ‘Wernicke’s syndrome’ or ‘Lewy bod*’ or DLB or LBD or ‘cerebrovascular dis*’ or ‘cognitive disorder’ or ‘cognitiv* degeneration’ or ‘brain degenerat* or ‘parkinson* dement*’ or PDD or ‘behavioural variant frontotemporal dement’ or BvFTD or bv-FTD.

The above does not constitute an exhaustive list and a complete list of search terms used is available from the corresponding author. Results were then limited to human participants, peer-reviewed articles published between 2008 and June 2019. Designation of HIC or LMIC was based on classification by the Organisation for Economic Co-operation and Development (OECD, 2018). Where countries were designated LMICs with exemptions for certain areas or regions, the region was used to determine inclusion or exclusion. For example, China is currently categorised as LMIC but the special administrative regions of Hong Kong and Macao are categorised as HIC. As such, articles reporting on participants who resided in Hong Kong or Macao were excluded but articles reporting on participants who lived in other regions were included.

### Inclusion and exclusion criteria

Studies were included if:

1. They reported on a cognitive, psychological or social intervention.
2. The study population consisted of people with a formal diagnosis of dementia or probable dementia (any variant), using appropriate screening tools for the country in question.
3. The intervention was delivered as part of a quantitative or qualitative research programme.
4. The intervention was delivered in a country or region defined as being low- or middle-income (Organisation for Economic Co-operation and Development (OECD), 2018)

Studies were excluded if:

1. They reported on protocols or reviews.
2. They were not published in a peer-reviewed journal
3. The intervention was delivered to a family carer or healthcare professional only.
4. They reported on physiological interventions, invasive procedures, medicinal products and procedures, complementary or alternative medicinal procedures and products (e.g. exercise interventions, minor surgery, acupressure or acupuncture).

### Screening and selection

Search results were imported to EndNote, where duplicates were removed, and titles were screened against the eligibility criteria by the corresponding author. Following this, all abstracts were screened, and full text articles were sought for studies included. Full texts were then screened against the eligibility criteria by authors CS and ML independently. Decisions on inclusion or exclusion were made independently and discrepancies discussed. Reference lists of included articles were also searched to identify further studies for inclusion.

### Quality assessment

The quality of included studies was rated independently by two raters (HD and ML) using two checklists. The Downs and Black checklist is 27-item checklist designed to assess the quality of both randomised an non-randomised studies (Downs & Black, 1998). To assess how ready interventions were for implementation, the ImpRess was used (Table 1). The ImpRess is a 26-item checklist that covers ten implementation readiness themes including motivation, theory of change, implementation context, experience, manager support and resources (Streater, Spector, Aguirre, Stansfeld, & Orrell, 2016). A *κ* was then calculated to assess agreement between the two raters on both checklists.

**Table 1.** ImpRess checklist.

Theme (max score)	Question	Checklist
Motivation (/10)	Does the study describe why management decided to subject the employee population to the organizational change?	1. Does the existing evidence suggest the intervention is likely to be cost effective?
		2. Does the existing evidence suggest the intervention is likely to be effective for the primary outcome?
		3. Does the existing evidence suggest the intervention is likely to be effective for other key outcomes?
		4. Are there other benefits for the patient (qualitative)?
		5. Are there benefits for the organisation?
Theory of change (/8)	Was the intervention design influenced by a theory of change describing the proposed pathway from implementation to health outcome?	6. Are the outcomes clearly defined?
		7. Is how the intervention works clearly defined?
		8. Is the design suitable for the kind of intervention (RCT)?
		9. Is there a coherent theoretical base?
Implementation context (/4)	Does the study provide any useful contextual information relevant to the implementation of the intervention?	10. Is the intervention standardised?
		11. Can it be widely implemented in practice (following on from a research setting)?
Experience (/4)	Does the study establish whether those implementing the intervention had appropriate experience?	12. Are the skills and experience of the person delivering the intervention clearly described?
		13. Is there monitoring of the delivery (attendance/ adherence) of the intervention?
Planning consultations (/4)	Is there a report of consultation/ collaboration processes between managers, employees and any other relevant parties during the planning stage?	14. Is the amount of time necessary to set up the intervention specified?
		15. Is the planning and setting up of sessions clearly defined?
Delivery collaborations (/4)	Is there a report of consultation/collaboration processes between managers, employees and any other relevant parties during the delivery stage?	16. Does it specify the amount of time required for each session and for the duration of the programme?
		17. Are the potential barriers and facilitators to the delivery of the intervention described?
Manager support (/2)	Were on-site managers/supervisors supportive of the intervention?	18. Is the level of managerial support described during the intervention/evaluation?
Employee support (/2)	Were employees supportive of the intervention?	19. Is the level of support required by the staff members to deliver the intervention described?
Resources (/10)	Does the study give information about the resources required in implementing the intervention?	20. Are the resources required to deliver the intervention specified?
		21. Are the training costs specified?
		22. Are the training materials specified?
		23. Are there manuals for the intervention?
		24. Are the materials easy to source?
Population characteristics (/4)	Does the study provide information on the characteristics of the people for whom the intervention was beneficial, and the characteristics of those for whom it was harmful or ineffective?	25. Are the population characteristics specified?
		26. Does it specify who benefits most from the intervention?

2 = Criteria fully met; 1 = criteria partially met; 0 = no information given.

## Results

**Figure 1.**
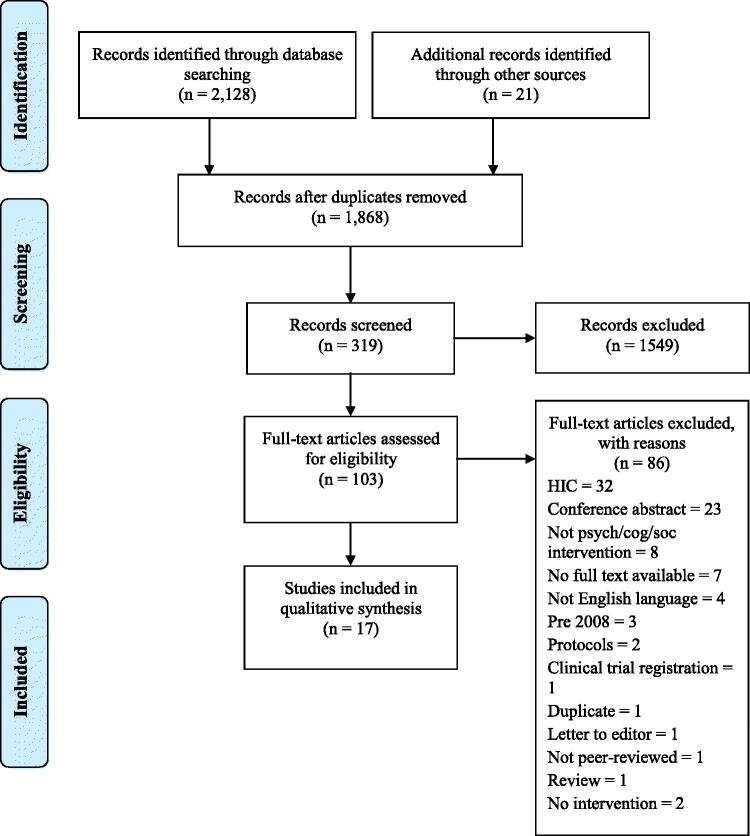
Article screening flow diagram.

A total of 1847 articles were identified after duplicates were removed. Title screening resulted in a further 1549 articles being excluded, leaving 298 for abstract screening. Abstract screening resulted in a further 216 articles excluded, leaving 82 for which full texts were sought. Of the 82 full texts, 23 were conference abstracts and no full text was available. Other reasons for exclusion included interventions were in HICs (*n* = 22), not cognitive, psychological or social interventions (*n* = 8), no full text available (*n* = 6), written in languages other than English that were not spoken by co-authors (*n* = 4), protocols (*n* = 2), clinical trial registration abstract, duplicate and letter to editor and no intervention (*n* = 4).This left 13 full texts included. An additional 21 studies were identified from references, with four ultimately included. Reasons for exclusion at this stage were: designated HIC (*n* = 10), study conducted over 10 years ago (*n* = 3), not peer-reviewed (*n* = 1), review (*n* = 1), no intervention (*n* = 1) and no full text available (*n* = 1). Full texts were screened for inclusion independently by CS and ML. The screeners mostly agreed on ratings, with the exception of two studies where the nature of the intervention was ambiguous. The screeners discussed these instances and ultimately decided upon excluding them as the intervention was not deemed psychological, social or cognitive. Consequently, 17 articles pertaining to 11 different interventions, in six countries were included in the current review (Figure 1).

### Quality assessment

As all included studies were underpowered or pilot studies, the last item of the Downs and Black checklist regarding sufficient power to detect a clinically important effect was omitted. Agreement between raters was *κ* = 69.7% for the Downs and Black checklist and κ = 81.9% for the ImpRess, indicating substantial agreement (Landis & Koch, 1977).

The average Downs and Black score awarded to studies was 19/26 (Table 2). The lowest quality study was the adaptation of CST in sub-Saharan Africa (12/26) (Mkenda et al., 2018) and the highest quality studies were cognitively stimulating activities intervention in China (Niu, Tan, Guan, Zhang, & Wang, 2010) and a Tailored Activity Programme in Brazil (Novelli et al., 2018) (both 24/26). Successful blinding of participants to allocation into case and control groups (item 14) was not possible due to the nature of the interventions. Also, all studies failed to report or measure adverse events and compliance to treatment or refusal rates were often not reported.

**Table 2.** Summary of included studies.

							Significant quantitative improvement (✓/✘/-)?			
Intervention	Duration	Country	Author	Participants (*n*)	Average downs & black rating (/26)	Average ImpRess rating (/52)	Cognition	Quality of life	ADLs	BPSD
Cognitively Stimulating Activities (CSA)	10 weeks	China	(Niu et al., 2010)	32	24	27	✓	–	–	✓
Cognitive Stimulation Therapy (CST)	7 weeks	India	(Raghuraman et al., 2017)	9	13	36	–	–	–	–
		Tanzania & Nigeria	(Mkenda et al., 2018)	16	12	28	–	–	–	–
		Tanzania	(Paddick et al., 2017)	34	23	35	✓	✓	–	✓
Reality Orientation (RO)	6 months	Brazil	(Camargo, Justus, & Retzlaff, 2015)	14	16	19	✓	–	–	–
Reminiscence Therapy (RT)	24 weeks	Argentina	(Serrani Azcurra, 2012)	132	22	26	✘	✓	✘	✘
	12 weeks	Turkey	(Aşiret & Kapucu, 2016)	62	18	24	✓	–	✘	✓
Folk Recreation Programme (FRP)	16 weeks	China	(Li & Li, 2017)	40	19	23	✓	–	✓	✓
Go-Game	6 months	China	(Lin et al., 2015)	147	21	21	–	✓	✓	✓
Singing Intervention	3 months	China	(Lyu et al., 2018)	288	20	23	✘ (MMSE); ✓ (AVLT)	–	✘	✓
			(Wang et al., 2018)	60	16	19	✓	–	–	✓
Multidisciplinary Cognitive Rehabilitation Programme (MCRP)	12 weeks	Brazil	(Machado et al., 2009)	19	19	19	✘	✘	✘	✘
			(Viola et al., 2011)	41	20	25	✘	✓	–	✓
			(Santos et al., 2015)	97	21	26	✓	✓	–	✓
Occupational Therapy (OT)	5 weeks	India	(Kumar et al., 2014)	77	20	27	–	✓	–	–
Tailored Activity Programme (TAP)	3 months	Brazil	(De Oliveira et al., 2017)	21	23	30	–	–	–	✓
			(Novelli et al., 2018)	30	24	32	–	✓	–	✓

✓ = significant improvement, ✘ = no improvement, - = domain was not assessed/results not reported.

The average ImpRess score was 26/52. The intervention most implementation ready was a CST adaptation in India (36/52) (Raghuraman, Lakshminarayanan, Vaitheswaran, & Rangaswamy, 2017) and the least was a singing intervention in China (19/56) (Wang et al., 2018). Researchers commonly reported on factors outlining the motivation for choosing the intervention, the pathway to change impacted by the intervention from implementation to health outcome and characteristics of the populations that benefits from the intervention. However, no study reported training costs nor the amount of time necessary to set up the intervention. Many also failed to report on the nature of barriers and facilitators towards successful implementation as well as managerial and employee support required for implementation in most instances.

### Structured therapeutic interventions

#### Cognitively stimulating activities (CSA)

##### China

Scoring high on quality (24/26) and moderately on implementation readiness (27/52), thirty-two people with a diagnosis of probable Alzheimer’s disease and taking anticholinesterase inhibitors were recruited from a military sanatorium and randomly allocated to either CSA or control (Niu et al., 2010). Those in the CSA group individually completed a series of tasks. These were: a reality orientation task, a verbal and categorical fluency task, an overlapping figure naming task and a photo-story learning task. These tasks were administered individually, and difficulty was selected for each participant. People in the control group participated in a communication exercise where discussions about recent news topics and research on Alzheimer’s disease were held. After 10 weeks, a small improvement was noted for MMSE scores in the CSA group (0.81, SE = 0.16), whilst MMSE showed a small decline in the control group (0.19, SE = 0.16). Mann-Whitney *U* tests were used to compare change from baseline and suggested that CSA significantly reduced apathy (*Z* = –2.59, *p* = 0.017) and depression (*Z* = –2.437, *p* = 0.047) as measured by the Neuropsychiatric Inventory (NPI).

#### Cognitive stimulation therapy (CST)

##### India

Whilst scoring low on quality, the adapted, manualised CST intervention in Chennai was awarded the most points for implementation readiness, due to the thorough consideration of implementation factors including the motivation for and the climate or context in which implementation would take place. As part of the study, researchers culturally adapted, translated and tested the feasibility and acceptability of delivering CST for people who had been given a diagnosis of dementia (Raghuraman et al., 2017) using an established five stage procedure (Aguirre, Spector, & Orrell, 2014). Examples of implementation considerations were the provision of transport at a low fee and the provision of tea and biscuits at the start of sessions. Changes to CST sessions included adding a traditional South Indian counting game and, in place of meal planning and cooking activities, a meal budgeting activity was used. After adaptation, two pilot studies were conducted in which people with dementia were asked about their attitude toward the group, caregivers were surveyed for feedback and facilitators recorded feedback at the end of each session. Nine men were recruited to take part in the two CST pilots but three withdrew over the course of the intervention, leaving six people with dementia attending. Modifications were made to the manual based on findings from the first pilot and incorporated into the second pilot. For example, a group song sung at the beginning of each session was felt to be childish and replaced with popular local songs played in the background after the sessions. No formal or statistical analysis was conducted due to the small sample size. Participant feedback resulted in the session time being increased and caregiver feedback was positive, with some noting observable improvements in language, communication and quality of life. Facilitators noted that sessions were enjoyable but some sessions such as ‘being creative’ were challenging due to the perception of activities as childish by participants. Difficulties encountered included a potential language barrier with some participants preferring to converse in English rather than Tamil and the lack of female participants.

##### Tanzania and Nigeria

Two included articles pertained to the four-year Identification and Interventions for Dementia in Elderly Africans (IDEA) research programme. As part of IDEA (Paddick et al., 2015), researchers adapted and evaluated manualised CST for people with dementia. The adaptation and pilot (Mkenda et al., 2018) was conducted with people in rural settings in both Tanzania and Nigeria and, as with the Indian adaptation, followed an internationally established procedure for adapting CST to other countries and cultures (Aguirre et al., 2014; Hwang, 2009). Participants were identified from a previous dementia (all variants) prevalence study. Examples of implementation considerations included low literacy levels, transportation difficulties in rural settings, seasonal weather (i.e. rainy season) and the privacy and location of settings. Throughout the CST trial, Mkenda et al. (2018), also gathered data on key issues underpinning the provision of CST. For example, it is usually customary for older adults to be relieved of household responsibilities by younger relatives, but this contradicts CST principles of encouragement to engage in mentally stimulating activities. Other areas of implementation discussed were the prioritisation of village events, and the subsequent need for flexibility and contingency plans for sessions, and the preference for tangible treatment in place of psychological therapies. As part of the pilot, five older females from the Hai district of Tanzania and 11 older adults from Lalupon in Nigeria completed the full 14 sessions of CST. Whilst outcomes were collected, these were primarily used to train healthcare workers in outcome measurement and no analysis was reported. This meant that, whilst scoring 28 on the ImpRess checklist, the study was only awarded 12 for quality on the Downs & Black Checklist. Anecdotal feedback suggested that attendees found the group enjoyable and carers noted that their relative appeared more ‘more active and more interested in activities’ (Mkenda et al., 2018, p. 525).

Following the adaptation, CST was evaluated in a further stepped-wedge trial in the Hai district of Tanzania (Paddick et al., 2017). Participants were included if they met the Diagnostic and Statistical Manual (DSM-IV) criteria for dementia (all variants). The design of the trial meant that this particular study of CST was awarded a higher score for quality (23/26) and implementation readiness (35/52), indicating strong evidence for CST. Thirty-four people with dementia were allocated to three CST groups and three participants withdrew over the course of the study. All measures used were reported to be adapted or validated for use in Tanzania. Feasibility analysis indicated that attendance was 85% and sessions could be missed due to illness or family events. Furthermore, a question on the WHOQOL-Bref regarding sexual relationships was frequently omitted (69.6% of cases). Economic analyses indicated that the total cost of the CST intervention was 581,260 Tanzanian Shillings ($268) but formal healthcare costs per month did not differ prior to and following CST (*p* = 0.641). A significant improvement in cognition was noted on the adapted (Paddick et al., 2017) Alzheimer’s Disease Assessment Schedule Cognitive subscale (ADAS-Cog) from pre-CST (*M* = 29.3 SD = 8.134) to immediately post-CST (*M* = 23.1, SD = 7.597; *t* = 5.864, *p* < 0.001). There was a significant improvement from pre-CST (*M* = 11.4) to immediately post-CST (*M* = 13.1) on the WHOQOL-Bref Physical function subscale (*W* = 199, *Z* = 02.048, *p* = 0.041). There was also a significant reduction in the number of behavioural and psychological symptoms present and the severity and the distress this caused carers, as measured by the NPI. Specific behaviours were depressive symptoms, night-time disturbance and changes in appetite.

#### Reality orientation (RO)

##### Brazil

Described by Camargo, Justus, and Retzlaff (2015), participants who had been diagnosed with Alzheimer’s disease according to the DSM-IV, were either assigned to the intervention or treatment as usual (TAU) group based on their order of arrival at a neurology outpatient unit in Paraná, Brazil. However, both the quality (16/26) and implementation readiness (19/52) were low. Those receiving RO were invited to attend weekly sessions lasting between 30 and 60 minutes. Sessions consisted of continuous exposure to memory and orientation related information using several approaches. Participants were required to fill in personal information forms and forms relating the current weather and time. They also were required to learn the layout of rooms, including five reference objects. Lastly participants took part in an intensive dialogue session with a researcher and were encouraged to increase social engagement wherever possible. Fourteen people with Alzheimer’s disease were included in the trial, split evenly between RO and TAU and outcomes were collected every two months for six months after the RO sessions had begun. Results indicated that there were no significant differences between groups at initial follow-ups on the Consortium to Establish a Registry for Alzheimer’s Disease (CERAD; Fillenbaum et al., 2008) battery (*p* < 0.169) or on Clock Drawing Test (CDT; Sunderland et al., 1989) (*p* = 0.183). However, by the six-month follow-up, there was a significant improvement within groups (*M* = 35.57, *M* = 40.57, *p* < 0.037) on the CERAD but not the CDT (*p* = 0.183). Mean Mini Mental State Examination (MMSE) (Folstein, Folstein, & McHugh, 1975) scores also improved between groups (*p* = 0.039) and within groups for the experimental arm (*p* = 0.0018) at the second follow-up.

Specific implementation challenges were not considered by the authors. However, they did note that the novel use of the CERAD battery and significant findings indicated that RO may be a suitable intervention for people with dementia in Brazil but confirmation of the findings with a larger sample was needed.

#### Reminiscence therapy (RT)

##### Argentina

People with Alzheimer’s disease who resided in a long-term care facility were recruited for a single blind, RCT of a reminiscence intervention (Serrani Azcurra, 2012). The intervention consisted of 24 bi-weekly sessions, lasting one hour. Participants joined a peer group where coordinators offered memory triggers to promote personal and shared memories, with the assistance of a relative where appropriate. Common themes included childhood, employment, illnesses, marriages, parenthood and deaths. This was followed by a general discussion in which shared concepts were explored. An active control group attended counselling and had informal social contact bi-weekly for one hour at a time. A passive control group received unstructured social contact, again bi-weekly for one hour at a time. The study was rated 22/26 for quality and 26/52 for implementation readiness. A 3 × 3 multivariate analysis of covariance (MANCOVA) indicated a significant interaction effect of group and time at a 12-week follow-up and a six-month follow-up on the Self-Reported Quality of Life scale (SRQoL: Kane, 2003) and Social Engagement Scale (SES; Mor et al., 1995) (*T*_1_ (*F*(130, 2) = 0.641, *p* < 0.01, *η*^2^ = 0.1; *T*_2_ (*F*(130, 2) = 0.352, *p* < 0.01, *η*^2^ = 0.2). Within subjects analysis indicated no significant improvement on these measures in either control group but a significant improvement was documented in the intervention group for both the SRQoL (*F*(130, 2) = 0.217, *p* < 0.01, *η*^2^ = 0.26) and the SES (*F*(130, 2) = 0.225, *p* < 0.01, *η*^2^ = 0.08). No significant differences were noted for cognition, activities of daily living, well-being or caregiver burden.

##### Turkey

An additional reminiscence intervention (*n* = 62) was conducted in a long-term care facility for people with mild and moderate Alzheimer’s disease in Ankara (Aşiret & Kapucu, 2016), receiving 18/26 for quality and 24/52 for implementation readiness. Specific activities included group reminiscence on the following topics: childhood, family, school days, work life or daily life for those who did not work, fun days outside the home, marriage and celebrations. Sessions lasted between 30 and 45 min and were conducted weekly for 12 weeks. The control group received a conversation lasting between 20 and 25 min per week on current topics such as health or current issues. Following the intervention, participants in the intervention group had a higher MMSE score (*M* = 18.54, SD = 3.36) than those in the control group (*M* = 14.35, SD = 1.99; *p* < 0.001). GDS scores for the experimental group (*M* = 9.32, SD = 2.82) were significantly lower than the control group (*M* = 14.35, SD = 4.66; *p* < 0.001). A Daily Living Activities Observation Form developed for the study by the authors indicated an improvement in the median on communication, collaboration and socialisation (pre-test median = 33.3, post-test median = 66.7).

### Recreational interventions

#### Folk recreation programme (FRP)

##### China

Scoring moderately on both quality (19/26) and implementation readiness (23/52), people with dementia (all variants) were recruited from a long-term care facility in Hangzhou and received the intervention three times a week for 16 weeks. Each session lasted 40–50 min (Li & Li, 2017). Examples of folk activities included arts, games and music. Art activities were mainly based on Chinese tales or traditional festivals. Games were usually upper body activities and, for the music activities, folk songs that were easy to sing were selected. Participants allocated to the experimental group also received one-to-one interventions two times a week and lasting 30 min a session. A total of 48 participants were recruited and evenly split between the intervention and TAU. Five participants withdrew from the experimental group. After 16 weeks, average scores on the MMSE (*M* = 14.58, SD = 5.59; *M* = 17.00, SD = 4.03, *p* < 0.001) and the Barthel Index (*M* = 77.37, SD = 11.95; *M* = 90, SD = 3.73, *p* < 0.001) had significantly improved and scores on the Chinese version of the NPI had significantly decreased. Specifically, dysphoria (*p* < 0.001), delusions (*p* = 0.02) and apathy (*p* = 0.04) were significantly lower at post-test.

#### GO-Game (chinese chess)

##### China

Go-Game or ‘Baduk’ is a form of Chinese chess and (Kim et al., 2014), in a RCT in Shenyang, 147 people with Alzheimer’s disease only were randomised to one of three groups: control (*n* = 49), short GO intervention (one hour daily, *n* = 49) or long GO intervention (two hours daily, *n* = 49) (Lin, Cao, & Gao, 2015) over a six-month period. The study scored moderately on both quality (21/26) and implementation readiness (21/52). No participants had prior experience of the game and were invited to learn the rules via Wikipedia. Despite an average age of 42.17, the mean Mini Mental State Examination (Folstein et al., 1975) score (MMSE) at baseline was 19.2. At follow-up, intervention groups reported significantly less depressive symptomology (control group: *M* = 16.73, SD = 9.46; short GO: *M* = 13.32, SD = 8.25; long GO: *M* = 12.10, SD = 8.25; *t* = 2.31, *p* = 0.02), as measured by the Montgomery and Asberg Depression Rating Scale (MADRS) (Montgomery & Åsberg, 1979) and significantly less anxiety (control group: *M* = 8.07, SD = 4.26; short GO: *M* = 6.59, SD = 4.11; long GO: *M* = 5.89, SD = 3.34; *t* = 2.22, *p* = 0.03), as measured by the anxiety subscale of the Hospital Anxiety and Depression Scale (HADS) (Zigmond & Snaith, 1983). Improvement was also noted on the Global Assessment of Functioning Scale (GAFS) (American Psychiatric Association, 2000) (*p* = 0.03) and the 36 item Short Form Health Survey for quality of life (SF-36) (Ware & Sherbourne, 1992) (*p* = 0.04). No improvement was noted for alexithymia (*p* = 0.46). No post hoc comparisons were conducted to assess differences between short GO and long GO.

#### Singing interventions

###### China

In a RCT of 298 people with Alzheimer’s disease, people were either assigned to a singing group (Group A) referred to as music therapy by the authors (Lyu et al., 2018), a lyric reading group (Group B) or to TAU (Group C). Group A were encouraged to sing songs picked by musicians and designed to be reminiscent of their 20s and 30s and Group B read lyrics to these songs. Both groups consisted of five or six participants and one therapist, and sessions ran twice daily for three months. Ten participants withdrew during the study period (Group *A* = 3, Group *B* = 3, Group *C* = 4), leaving 288 for analysis. There were no significant differences between groups at follow-up on the MMSE and Barthel Index. For the World Health Organisation University of California-Los Angeles, Auditory Verbal Learning Test (WHO-UCL AVLT) (Maj et al., 1993), Group A scored higher than Group C (*p* < 0.05) but there was no significant differences between Group B and C at the second follow-up. Further analysis according to the stage of Alzheimer disease suggested that, for people with moderate or severe dementia, the singing group was associated with less caregiver distress as measured by the NPI (*p* < 0.05). The quality of this study was rated as 20/26 and the implementation readiness was 23/52.

Similar to the above trial, an additional singing intervention was conducted in the Heilongjian Province (Wang et al., 2018) with 60 people with mild Alzheimer’s disease. However, this trial scored lower for quality (16/26) and implementation readiness (19/52). For the intervention group, participants attended a treatment site with soft lighting and little noise. Songs were then selected for specific participants by the research team and played at 40 decibels. Participants were encouraged to sing the songs alongside the therapist, three times per day for between 30 and 50 min per session over the course of three months. Results indicated that both the MMSE and Montreal Cognitive Assessment (MoCA; Nasreddine et al., 2005) improved in both groups, but that this improvement was greater in the singing group (*p* = 0.003; *p* < 0.001). As with the cognition measures, scores on the NPI decreased in both groups but was significantly lower in the singing group (*p* < 0.01).

### Multi-component interventions

#### Multi-disciplinary cognitive rehabilitation programme (MCRP)

##### Brazil

Three included articles pertained to a multidisciplinary cognitive rehabilitation programme (MCRP) conducted in services around São Paulo, where intervention activities consisted of: cognitive rehabilitation, computer-assisted cognitive therapy, physical training, physiotherapy and cognitive stimulation with reading and games. Each activity lasted between 60 and 90 min but intervention sessions were five hours each, over a 12-week period. The first of the studies to evaluate this intervention was a mixed method, single blind trial of MCRP versus standard care for 19 people with mild to moderate Alzheimer’s disease (Machado et al., 2009). This study was awarded a score of 19 for both quality and implementation readiness. There was no significant effect of the intervention on the GDS, QoL-AD self-rated and QoL-AD proxy rated. For the MMSE, there was no significant difference between pre and post-test for the experimental group but, in the control group, there was a statistically significant decline between baseline (*M* = 23.9, SD = 3.6) and post-test (*M* = 22.6, SD = 3.3). For the qualitative component, participants wrote more about their quality of life after the intervention (13.6 words on average compared to 48.5 words) and a fifth theme ‘quality of life has changed’ was present in half of the participants, indicating the intervention may have influenced quality of life.

A wait-list, single blind controlled trial of the MCRP was used for 41 people with Alzheimer’s disease and their caregivers. This study was awarded slightly more for quality (20/26) and implementation readiness (25/52). Participants were assigned to one of four intervention groups running twice a week over 12 weeks, with a maximum of 12 people with dementia per group (Viola et al., 2011). Five participants withdrew over the course of the study. Whilst the intervention was not associated with significant improvement on the Short Cognitive Test (SKT; Pereira, Viola, Flaks, Forlenza, & Yassuda, 2009), there was a slight but statistically significant decline in the control group between baseline (*M* = 12.6, SD = 5.4) and follow-up (*M* = 13.8, SD = 5.5, *p* = 0.05), where an increase in scores indicates worsening. There was a significant improvement in depression symptoms as measured by the GDS from both a participant (baseline: *M* = 4.7, SD = 3.1; follow-up: *M* = 3.4, SD = 3; *p* = 0.001) and carer (baseline: *M* = 3.9, SD = 3.5; follow-up: *M* = 3.1, SD = 2.9; *p* = 0.02) perspective. There was also an improvement in quality of life from both a participant (baseline: *M* = 35.2, SD = 5; follow-up: *M* = 37.3, SD = 4.4; *p* = 0.004) and carer (baseline: *M* = 30.8, SD = 5.2; follow-up: *M* = 33, SD = 6; *p* = 0.04) perspective.

A further single blind trial of this intervention with 62 people with mild Alzheimer’s disease was also conducted (Santos et al., 2015), and which scored 21 for quality and 26 for implementation readiness. Participants who had received the intervention showed a small but significant increase in MMSE scores from baseline (*M* = 23, SD = 2.5) to post-test (*M* = 23.6, *p* = 0.021), a significant decrease in depressive symptoms from baseline (*M* = 5.1, SD = 3.3) to post-test (*M* = 3.7, SD = 3, *p* < 0.001), as measured by the Geriatric Depression Scale (GDS; Paradela, Lourenço & Veras, 2005, Yesavage et al., 1982) and a significant increase from baseline (*M* = 34.9, SD = 6.3) to post-test (36.5, SD = 5.2, *p* = 0.003) for participant rated quality of life (QoL-AD; Novelli, Nitrini, & Caramelli, 2010). No significant differences were apparent for the control group or for carer rated quality of life in both the control and experimental group. Furthermore, there were no significant differences between baseline and endpoint for people with moderate dementia.

#### Occupational therapy (OT)

##### India

An OT intervention developed in New Delhi consisted of 10 sessions, lasting 70 min over a five-week period (Kumar et al., 2014). It was awarded 20/26 for quality and 27/52 for implementation readiness. Activities included muscle relaxation, physical exercises, self-care activities, cognitive exercises and recreational activities. The sessions were facilitated by a trained occupational therapist. In a RCT of 77 people with dementia (all variants) results indicated a significant improvement in quality of life from baseline (*M* = 66.78, SD = 3.68) to follow-up (*M* = 71.36, SD = 4.66, *p* < 0.001). As assessed by the Hindi version of the WHOQOL-Bref, (Saxena, Chandiramani, & Bhargava, 1997) specific quality of life domains including physical, environmental, psychological and social often decreased in the control group but improved in the experimental group. Specifically, statistically significant improvements were noted for physical (*p* < 0.001), environmental (*p* = 0.006) and psychological (*p* < 0.001) aspects of quality of life in the experimental group.

#### Tailored activity programme (TAP)

*Brazil.* TAP was designed to both reduce behavioural and psychological symptoms of dementia (BPSD) and to reduce caregiver burden in an outpatient setting (TAP-O) over a three-month period (one session per week). Intervention activities consisted of eight sessions in which occupational therapists assessed a person with dementia’s abilities and interests, planned tailored activities, educated carers and encouraged implementation of meaningful activities to daily life, whilst control participants received psychoeducation (De Oliveira et al., 2017).

Two articles identified pertained to assessments of the TAP-O intervention in São Paulo. First, a RCT of 21 people with dementia (all variants) and carers resulted in a statistically significant decrease in hallucinations (*p* = 0.04), agitation (*p* = 0.03), anxiety (*p* = .02), aggression (*p* = .01), sleep disorder (*p* = .02), aberrant motor behaviour (*p* = 0.02) (De Oliveira et al., 2017) as measured by the Brazilian version of the Neuropsychiatric Inventory-Clinician rated (NPI-C: Stella et al., 2013) and carer burden (*p* = 0.003) as measured by the Brazilian Zarit Burden Scale (Taub, Andreoli, & Bertolucci, 2004). Adaptation from the original American version (Gitlin et al., 2009) was discussed, with authors using translation and back translation for the protocol, with a subsequent review by an occupational therapist. In addition, some modifications were made to ensure that the intervention could be carried out in an outpatient setting rather than the home. The quality rating for this study was 23/26 and implementation readiness was 30/52.

Second, a parallel two group, single blind RCT was conducted with 30 dyads, who had been diagnosed with dementia (all variants), in the community (Novelli et al., 2018). This study was of greater quality (24/26) and judged to be more implementation ready (32/52). Treatment fidelity was strengthened for this study through training for the occupational therapists delivering the intervention. Training lasted 24 hours in total and involved lectures and role-play sessions by an accredited TAP trainer. Interventionists were also closely supervised by the site co-ordinators and participated in bi-weekly meetings to troubleshoot implementation challenges. At the four-month follow-up, carers reported significantly greater quality of life (*M* = 41.47, SD = 4.07) than at baseline (*M* = 38.67, SD = 5.64; *p* = 0.02), whilst people with dementia reported no improvement in their own quality of life. There was a significant decrease in the total behavioural and psychological symptoms present from baseline (*M* = 6.53, SD = 3.96) to post test (*M* = 3.67, SD = 1.8; *p* < 0.001), the frequency of these behaviours (baseline: *M* = 15.07, SD = 5.84; post-test: *M* = 9, SD = 4.87; *p* < 0.001), the intensity of these behaviours (baseline: *M* = 8, SD = 4.31; post-test: *M* = 5.07, SD = 3.24; *p* < 0.001) and caregiver distress caused by these behaviours (baseline: *M* = 13.63, SD = 9.65); post-test: *M* = 6.87, SD = 5.15; *p* < 0.001), as measured by the NPI. There were no significant differences on the Zarit Burden Scale between baseline (*M* = 30.33, SD = 11.44) and post-test (*M* = 30.4, SD = 15.39; *p* = 0.5). There were significant interaction effects for group x time for proxy quality of life (*p* = 0.006), carer quality of life (*p* = 0.004), NPI total (*p* = 0.016), frequency of NPI behaviours (*p* = 0.020), intensity of NPI behaviours (*p* = 0.025), caregiver distress as measured by the NPI (*p* = 0.002). There was no significant interaction for burden (*p* = 0.321).

## Discussion

Seventeen articles describing 11 different interventions were identified. Interventions were documented in Argentina, Brazil, China, India, Nigeria, Tanzania and Turkey. The quality of included studies was variable, as were the interventions and outcomes used. Most notably, the highest score awarded for implementation readiness was 36/52, with almost all studies failing to consider or report levels of managerial or employee support for the intervention. Whilst this may be novel from a research perspective, from an implementation perspective it is of vital importance. Commitment, involvement and accountability of managers is an essential component of a conducive implementation climate and increases the likelihood of successful implementation of an intervention (Damschroder et al., 2009). Other implementation factors including the level of staff experience, consultations with relevant parties and the resources needed were considered sparingly.

The ImpRess checklist was initially developed for assessing the implementation readiness of CST in the UK. Whilst the criteria may be overly ambitious for countries where psychosocial research is in earlier stages, it was nonetheless a feasible means of comparing the implementation readiness of psychosocial interventions in general and in different healthcare systems.

Populations either consisted of people with a diagnosis of Alzheimer’s disease only or those who had been diagnosed with dementia of any variant. Of those where the study population was limited to those with Alzheimer’s disease, it was not always clear whether additional methods such as imaging were used to confirm this diagnosis. Further, of the studies that included people with unspecified dementia, no sub-analysis based on variant were included. Thus, it is not possible to determine whether the interventions documented here have varying effectiveness based on the type of dementia people have been diagnosed with.

Most interventions were comprised of a combination of psychological, social and cognitive activities and evidence for specific outcomes associated with these were variable. For cognition, seven of the 11 interventions were associated with significant improvement. These were cognitively stimulating activities, a folk recreation programme and singing intervention in China, a group CST programme in Tanzania, a Multidisciplinary Cognitive Rehabilitation Programme and Reality Orientation intervention in Brazil and Reminiscence Therapy in Turkey. Quality of life was assessed in eight studies, with seven noting an improvement. Of all the studies here, two reported both an increase in cognition and an increase in quality of life: CST and MCRP. However, of these, CST was considered more implementation ready due to its consistently higher scores on the ImpRess checklist. Furthermore, CST was the only effective intervention to have been used in three countries, with MCRP lacking evidence outside of Brazil. CST is a manualised intervention for which there is an established procedure for adaptation to different countries and cultures (Aguirre et al., 2014). From an implementation perspective, a culturally appropriate adaptation increases the relevance of an intervention and subsequently may increase the likelihood that the intervention is scaled up for practice. Combined with the quantitative and qualitative evidence found here, there appears to be a strong evidence base for CST. Thus, of the interventions described here, CST is recommended as an effective and implementation ready intervention that can be used successfully in LMICs.

### Methodological problems and limitations

The search strategy was designed to capture all psychological, social and cognitive interventions in use for people with dementia in LMICs. Whilst the interventions included were varied, the descriptions of exactly what activities these interventions entailed could be vague and this hampered decision making when including and excluding results. In addition, some interventions were referred to as a specific therapy but, upon closer examination of the activities the intervention entailed, it was not clear whether this was the case. For example, CST was developed in the UK as a manual based, non-pharmacological intervention for people with mild to moderate dementia (Spector, Orrell, Davies, & Woods, 2001). However, despite referencing the original CST research and referring to their intervention as CST, the authors developed a new programme of tasks designed to stimulate executive function and working memory (Niu et al., 2010). As this was not a study of manualised CST, the programme has been referred to as Cognitively Stimulating Activities (CSA) here.

Related to this was the inclusion of two singing interventions in China. Music therapy in practice is diverse but generally refers to interventions in which the use of music and its elements are used promote an individual’s health and wellbeing (McDermott, Crellin, Ridder, & Orrell, 2013). Despite referring to the intervention as ‘music therapy’, it may be more accurate to describe these interventions as recreational singing groups, due to the absence of any additional music therapy techniques (McDermott, Ridder et al., 2018).

There was wide heterogeneity amongst the studies included here. Intervention activities were primarily designed to target specific outcomes such as quality of life or cognition and, of those that targeted cognition, it was often different cognitive functions. Further, interventions often vary in dose, with the shortest intervention time documented here as seven weeks and the longest six months. As such, comparisons across psychosocial interventions can be problematic, limiting any recommendations given.

Most measures used in included studies were standard for dementia research. Namely, authors used measures of cognition, quality of life, activities of daily living (ADL), and behavioural and psychological symptoms of dementia (BPSD). However, the specific measures and quality of these measures were variable. For example, the MMSE was used most commonly in 9 studies, but other measures of cognition included the MoCA, the ADAS-Cog, the CERAD and the Short Cognitive Test. All cognition measures used were developed in Western countries and the psychometric properties of the measures in the different countries were not reported. This is important given that Western measures of cognition can have a high false positive rate when used in some LMICs (Paddick et al., 2017).

### Future research

As mentioned, measures included in studies here were predominantly developed in Western countries. To ensure that recommendations are based on a robust evidence-base, researchers may wish to explore the quality of measures when they are used in intervention research in LMICs.

Interventions that focused only on a caregiver were excluded as the aim of the review was to identify interventions where people with dementia were the primary target population. This meant that, although research pertaining to dyads was included, articles from notable programmes of research in LMICs such as those by the 10/66 Dementia Research Group (e.g. Guerra, Ferri, Fonseca, Banerjee, & Prince, 2010) were often excluded. Future researchers may wish to explore the effectiveness of carer programmes for people with dementia.

The MCRP in Brazil also produced a significant effect on both cognition and quality of life and addressed some implementation factors. There is good evidence for the effectiveness of this intervention but evidence in LMICs outside of Brazil is lacking, meaning it cannot yet be recommended for use in different countries. A thorough examination of implementation and cultural adaptation would address this and provide evidence for its effectiveness outside of Brazil.

Very few studies included in this review reported on the implementation aspects of introducing interventions across countries and cultural contexts. Future research on the effectiveness of psychological, cognitive and social interventions in the LMIC context would benefit from the routine inclusion of implementation readiness data. This will inform clinical practice and policy, aiding the scaling up of interventions to widespread implementation.

## Conclusion

A systematic search of databases was used to identify 11 different psychological, social or cognitive interventions in use for people with dementia in LMICs. Interventions and quality were variable but, based on assessments of both efficacy and implementation readiness documented here, CST appears to be a suitable intervention for people with dementia in some LMICs. This is due to documented improvements to both improve quality of life and cognition and a thorough consideration of implementation across countries. Other interventions including the MCRP and Go-Game were judged similarly effective but lacked data on implementation readiness outside of their country of origin.
